# Toward a Comprehensive Understanding of Cation Effects in Proton Exchange Membrane Fuel Cells

**DOI:** 10.1021/acsami.2c07085

**Published:** 2022-07-26

**Authors:** ChungHyuk Lee, Xiaohua Wang, Jui-Kun Peng, Adlai Katzenberg, Rajesh K. Ahluwalia, Ahmet Kusoglu, Siddharth Komini Babu, Jacob S. Spendelow, Rangachary Mukundan, Rod L. Borup

**Affiliations:** † Material Synthesis and Integrated Devices Group, Los Alamos National Laboratory, Los Alamos, New Mexico 87545, United States; ‡ Energy Systems Division, Argonne National Laboratory, Argonne, Illinois 60439, United States; § Energy Technologies Area, Lawrence Berkeley National Laboratory, Berkeley, California 94720, United States

**Keywords:** proton-exchange membrane fuel cells, cation contamination, platinum alloy catalysts, durability, conductivity, mass transport, impedance modeling

## Abstract

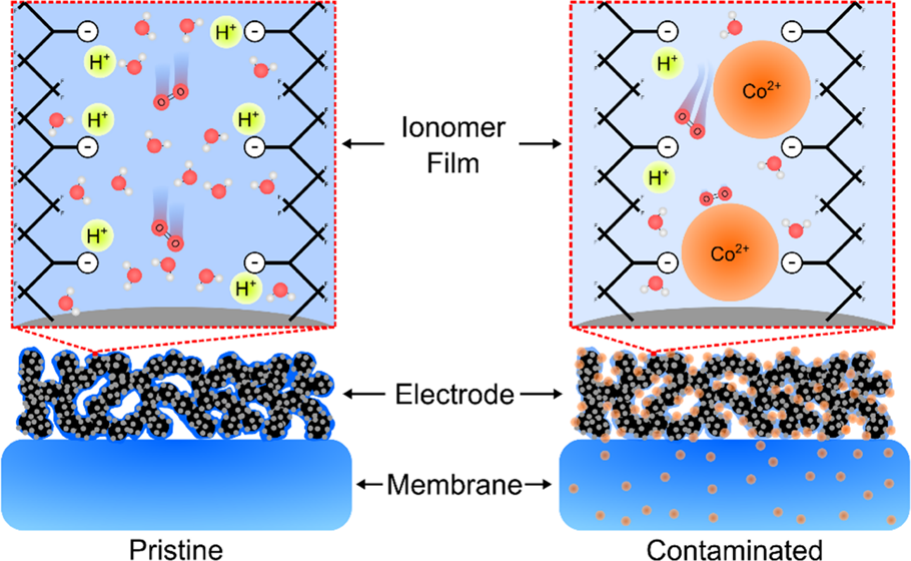

Metal alloy catalysts (e.g., Pt–Co) are widely used in fuel cells for improving the oxygen reduction reaction kinetics. Despite the promise, the leaching of the alloying element contaminates the ionomer/membrane, leading to poor durability. However, the underlying mechanisms by which cation contamination affects fuel cell performance remain poorly understood. Here, we provide a comprehensive understanding of cation contamination effects through the controlled doping of electrodes. We couple electrochemical testing results with membrane conductivity/water uptake measurements and impedance modeling to pinpoint where and how the losses in performance occur. We identify that (1) ∼44% of Co^2+^ exchange of the ionomer can be tolerated in the electrode, (2) loss in performance is predominantly induced by O_2_ and proton transport losses, and (3) Co^2+^ preferentially resides in the electrode under wet operating conditions. Our results provide a first-of-its-kind mechanistic explanation for cation effects and inform strategies for mitigating these undesired effects when using alloy catalysts.

## Introduction

The transportation sector is a major contributor to global CO_2_ emissions and is projected to contribute up to 27% of CO_2_ emissions by 2050 if no changes are implemented.^1^ Proton exchange membrane fuel cells (PEMFCs) are a promising alternative to internal combustion engines due to their ability to produce electricity on-demand without any local CO_2_ emissions. Despite significant technical progress, cost and durability remain major barriers to the wider adoption of PEMFCs.^2−11^ The ongoing development of advanced Pt-based alloy catalysts (e.g., Pt–Co, Pt–Ni) with improved oxygen reduction reaction kinetics holds great promise for enhanced PEMFC performance and reduced cost.^12−15^ The incorporation of alloying elements such as Co weakens the binding strength of oxygenated intermediates on the catalyst surface and facilitates dissociation of the double bond in O_2_ molecules, which consequently reduces the activation energy for the oxygen reduction reaction.^16^ A lower activation energy leads to a higher power density at a fixed Pt loading (≈0.16 g_Pt_·kW^–1^ reported in the literature^12^), allowing a decrease in the number of cells in a stack system and thus a reduction in the overall system cost. The practical application of the Pt–Co catalyst is further evidenced by its recent usage in the Toyota Mirai.^5,17,18^ However, for the wider adoption of Pt–Co catalysts, issues related to durability need to be addressed,^19,20^ which is particularly important for the emerging applications of PEMFCs in heavy-duty trucks.^5^

While Pt–Co catalysts can provide improved performance at the beginning of life, leaching of Co^2+^ from the catalyst and subsequent uptake in the electrode ionomer and/or membrane can cause performance degradation, particularly at high-current-density operations.^21^ As bulk Co segregates to the surface during the PEMFC operation, the surface Co becomes easily prone to leaching.^22^ O’Brien et al.^23^ measured a Co loss of 3.4 μg_Co_·cm^–2^ (initially 7.1 μg_Co_·cm^–2^) for Pt–Co supported on high-surface-area carbon and 3.6 μg_Co_·cm^–2^ (initially 11.2 μg_Co_·cm^–2^) for Pt–Co supported on Vulcan after 30 000 cycles of a catalyst-accelerated stress test (AST) for 0.1 mg_Pt_·cm^–2^ loading. The leached Co (i.e., Co^2+^) ion-exchanges with the sulfonic acid functional groups (SO_3_^–^H^+^) in the electrode ionomer, which can increase the kinetic and mass transport losses.^24−28^ Specifically, kinetic losses can increase due to a loss in catalyst mass activity from Co leaching and an associated increase in the proton transport resistance of the ionomer in the cathode.^29^ Moreover, O_2_ transport losses can increase due to the reduction in the hydrophilic domain volume of the ionomer reducing the O_2_ permeability.^28^ Despite these undesired effects reported in previous studies, a systematic study of Co^2+^ contamination of the electrode ionomer has not been reported.

Our understanding of Co^2+^ contamination effects remains severely limited since previous studies have experimentally analyzed the effect of the Co^2+^ content on the performance by contaminating the membrane or entire catalyst-coated membranes (CCMs),^21,30,31^ whereas the cations originate from the cathode catalyst layer. Additionally, Co^2+^ is mobile,^31^ which necessitates a careful design of materials and experimental conditions to derive useful insights into the effect of Co^2+^ contamination. For example, Cai et al.^31^ used in situ synchrotron X-ray fluorescence analysis (XRF) to show that Co^2+^ distribution is strongly dependent on the cell design, and Co^2+^ migrates in both the in- and through-plane directions in the membrane electrode assembly (MEA). Additionally, modeling by Weber and Delacourt^32^ indicated that the membrane thickness and operating conditions have strong effects on the acceptable cation exchange in the membrane. Previous studies focusing on other cations in membranes also documented the key role of fractional cation exchange in altering the properties of membranes.^33−35^ These insights suggest that a carefully designed and systematic study of Co^2+^ contamination is a necessary first step in understanding and implementing strategies for mitigating Co^2+^-induced performance loss.

Here, we investigate the effect of the selective Co^2+^ contamination of the electrode in a PEMFC. First, we correlated our electrochemical results with two-dimensional (2D) XRF measurements to elucidate the underlying mechanism of changes in performance induced by Co^2+^ contamination. Then, we characterized the effect of Co^2+^ doping on perfluorosulfonic acid (PFSA) properties by conducting ex situ measurements of membranes doped with different Co loadings to provide an experimental explanation of the observed trends in electrochemical performance. Lastly, we conducted impedance modeling to deconvolute the changes in ohmic, kinetic, and mass transport resistances. Our impedance modeling results are coupled with ex situ measurements of the PFSA properties to estimate how much of the Co^2+^ ends up in the electrode and the membrane during our experiments. For details on Co doping, electrochemical testing, ex situ measurement, and modeling procedures, the readers are referred to the Methodology section. To the best of our knowledge, this work provides (1) the first quantitative assessment of Co^2+^ contamination effects in PEMFCs, (2) the first determination of allowable Co^2+^ contamination levels, and (3) the first quantitative estimation of Co^2+^ partitioning between the electrode and the membrane. Our approach also provides a robust platform for investigating other cation contamination phenomena in fuel cell electrodes (e.g., cations leached from metal bipolar plates^36^), as well as other electrochemical devices that utilize PFSA ionomer/membranes.

## Results and Discussion

### Cation Effects on Electrochemical Performance

**Figure 1:**
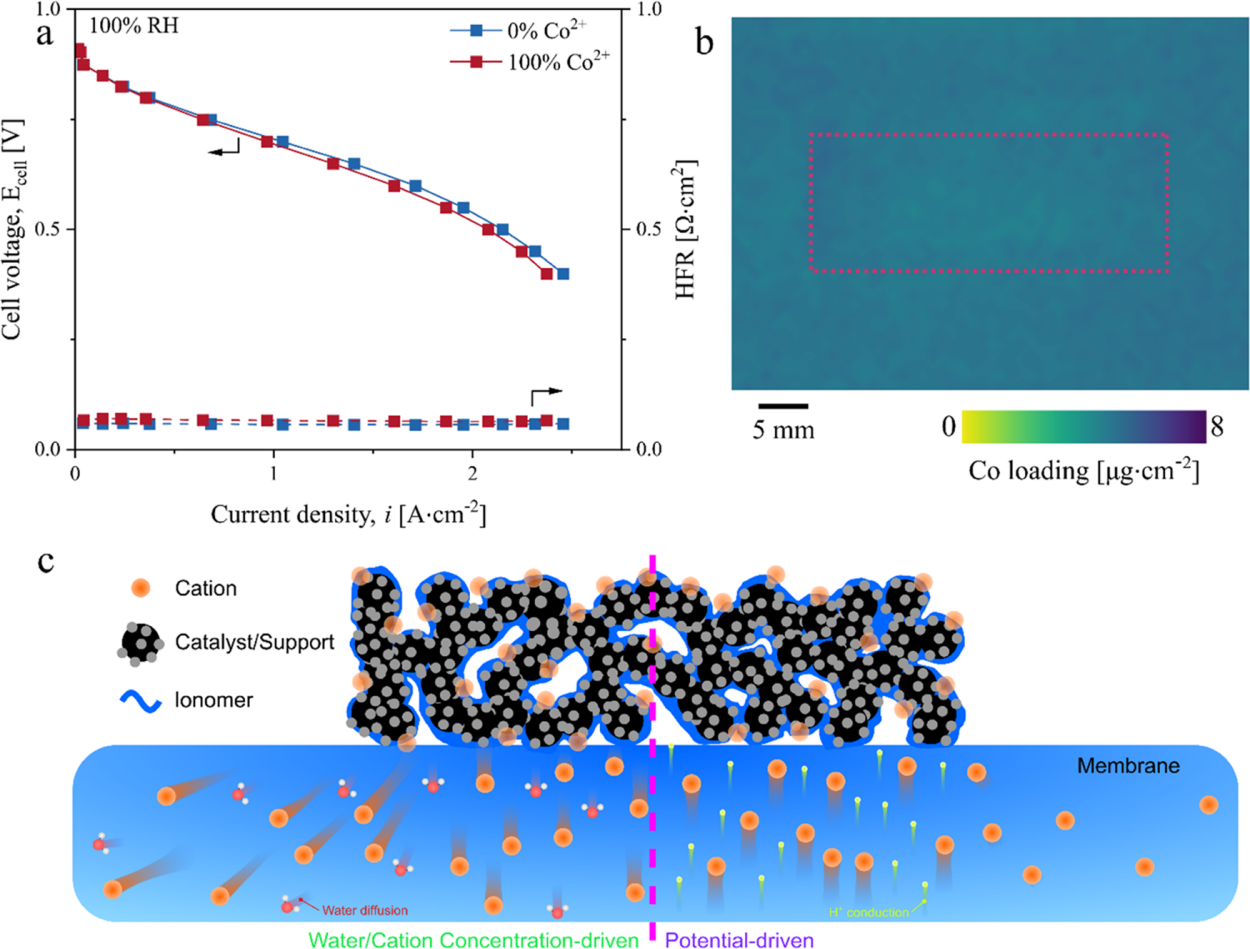
Co^2+^ doping effect on the performance of MEA with a large inactive membrane. (a) Polarization curves of MEA with a 100 cm^2^ membrane area showing a negligible effect of Co^2+^ doping on the performance. 50% RH data are shown in Figure S3. (b) 2D XRF analysis of 100% Co^2+^ MEA after testing. The average Co loading in the active area decreased from 11.0 to 3.2 μg_Co_·cm^–2^. The red-dashed-line rectangle indicates the active area. (c) Schematic showing two competing cation transport mechanisms: water/cation concentration-driven and potential-driven transport. The inactive membrane area acts as a Co^2+^ sink enabled by concentration-driven transport, leading to the negligible effect of Co^2+^ on performance. The schematic is not to scale.

Our initial investigation was on the effect of the selective Co^2+^ doping of the cathode electrode (5 cm^2^) on the performance, with a 100 cm^2^ membrane (N211) area that spans the entire area of the flow field (see Figure S1). The readers are referred to the Methodology section for details on the MEA preparation procedure. We found that the influence of the Co^2+^ concentration on performance was negligible (a decrease of 80 mA·cm^–2^ at 0.7 V), despite the electrode ionomer being 100% Co^2+^-exchanged (Figure 1a). To elucidate the cause of the negligible change in performance, we performed 2D XRF of the MEA after testing the 100% Co^2+^-exchanged sample (Figure 1b). Although the initial loading was 11.0 μg_Co_·cm^–2^, we observed only a 3.2 μg_Co_·cm^–2^ loading in the active area region after testing. This significant decrease in the loading was accompanied by an increase in Co loading from 0 to 2.8 μg_Co_·cm^–2^ in the inactive membrane area. We also verified that the Co^2+^ remained in the active area after transferring the electrode from the decal substrate to the membrane (Figure S2), meaning that the observed Co^2+^ migration predominantly occurred during cell operation. Additionally, we used inductively coupled plasma mass spectroscopy and verified that the exhaust water condensate did not contain detectable Co. Finally, we verified that Co^2+^ did not migrate into the polyurethane gaskets through XRF measurements. All of these results indicate that the Co^2+^ was leaving the cathode electrode and moving into the inactive membrane area, despite the applied potential promoting Co^2+^ retention in the electrode.^31^

In contrast to the dominating effect of potential-driven cation transport in the through-plane direction (i.e., the direction perpendicular to the membrane), the transport mechanism in the in-plane direction (i.e., the direction parallel to the membrane) is insensitive to the potential. However, cations can also diffuse in the membrane,^37^ and the cation diffusivity is directly proportional to the water content in the membrane.^38^ Since water (either generated or introduced to the cell via inlet gas streams) will diffuse from the active to the inactive membrane area, the inactive membrane area that is hydrated can act as a Co^2+^ sink (Figure 1c). To verify our hypothesis, we examined two MEAs with their cathodes 100% Co^2+^-exchanged but with a nonstandard conditioning procedure. Specifically, we operated one MEA under 100% RH with 20 polarization curves from 0.4 to 0.95 V and the other MEA at 50% RH (without any potential holds lower than 0.4 V) and performed XRF measurements of the MEAs after testing. Since the conditioning procedure included flooded operating conditions,^39^ we removed any low potential holds (i.e., <0.4 V) from this experiment. We observed that the Co^2+^ content in the active area after 100% RH testing was ∼52% lower compared to that after 50% RH cycling (4.3 and 8.9 μg_Co_·cm^–2^, respectively). Our findings support our hypothesis that hydration of the inactive membrane area promotes Co^2+^ migration in the in-plane direction, allowing the inactive membrane area to function as a Co^2+^ sink.

Our results thus far demonstrate that the hydration of membrane outside of the active area leads to the migration of Co^2+^ away from the active area, obscuring the effects of Co^2+^ contamination on cell performance. However, in a practical PEMFC system, the inactive membrane area is minimized to reduce the cost. To characterize how Co^2+^ contamination can affect cell performance under realistic fuel cell designs, the inactive membrane area needs to be reduced. We modified our MEA design, where we reduced the membrane area to 8.6 cm^2^ (N211) and used Kapton subgaskets to ensure a good seal (Figure S3 shows the membrane area relative to the active area). The crossover current was measured to be <2 mA·cm^–2^ across all MEAs with a minimized inactive area, verifying that the crossover current is consistent with the active membrane area. The readers are referred to the Methodology section for details on the MEA preparation procedure.

**Figure 2:**
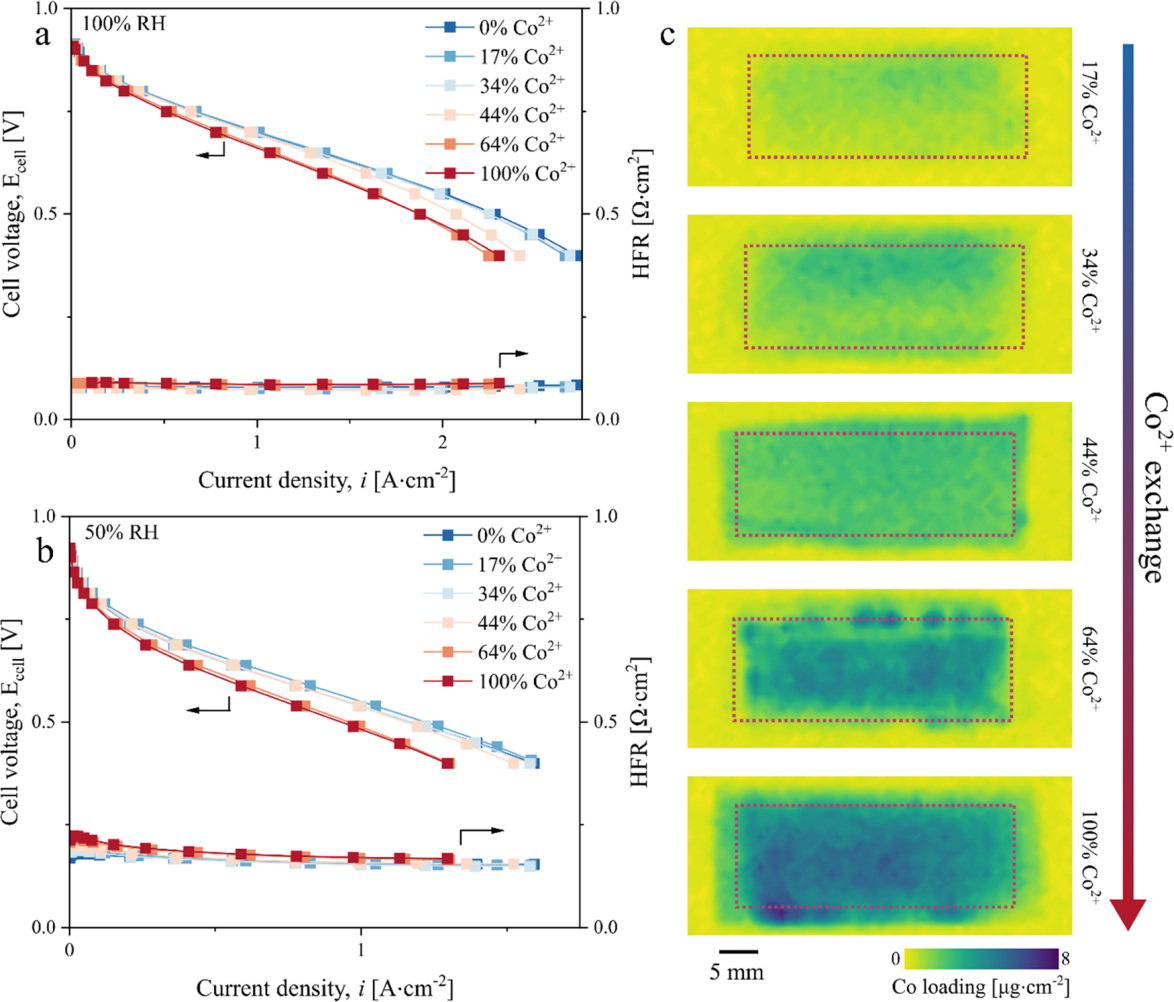
Co^2+^ doping effect on the performance of MEA with a minimized inactive membrane. (a, b) Polarization curves of MEA with an 8.6 cm^2^ membrane area showing a more pronounced effect of Co^2+^ doping on the performance at both 100% RH (a) and 50% RH (b). (c) 2D XRF measurements of 17, 34, 44, 64, and 100% Co^2+^ exchange (top to bottom). The average Co loading in the active area (red-dashed rectangle) is 1.6, 2.6, 3.7, 5.0, and 7.6 μg_Co_·cm^–2^.

We observed a stronger effect of Co^2+^ on the performance of MEAs with reduced inactive area in both wet and dry conditions (Figure 2a,b). The decrease in performance was accompanied by the relatively immobilized Co^2+^ in the active area (Figure 2c) compared to the MEA with a large inactive membrane area (Figure 1b). The MEA with 100% Co^2+^-exchanged cathode showed a post-testing Co loading of 7.6 μg·cm^–2^ in the active area, which is ∼2.4 times greater than that of the MEA with a large inactive membrane area. These results demonstrate that our proposed method can effectively suppress the Co^2+^ from leaving the active area.

**Figure 3:**
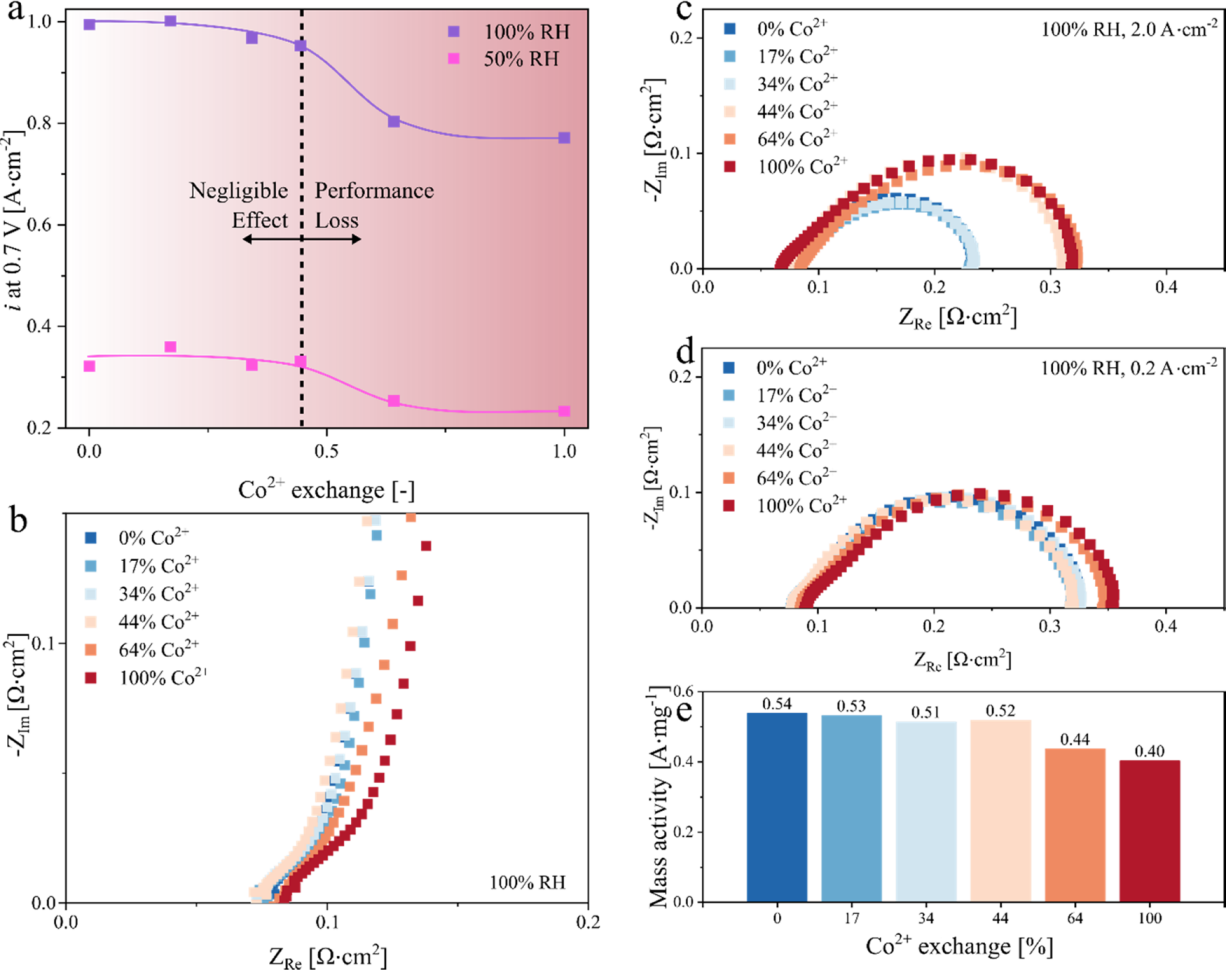
Electrochemical characterization of performance loss induced by Co^2+^ doping. (a) Change in current density with increasing Co^2+^ exchange at 0.7 V. The lines are sigmoidal fits for a visual aid of the reader. A decrease in performance at a 44% Co^2+^ exchange is accompanied by (b) an increase in proton transport resistance in the cathode electrode and (c) an increase in mass transport losses indicated by larger arcs of the electrochemical impedance spectra (EIS) spectra. (d) EIS spectra at low current density and (e) a decrease in mass activity also indicate potential changes in kinetic resistance. 50% RH EIS data are shown in Figure S4.

Comparing the performance at 0.7 V, the current density was observed to decrease by up to 23% at 100% RH and by up to 35% at 50% RH (Figure 3a). Interestingly, the performance remained relatively unchanged up to a critical Co^2+^ exchange of ∼44% and then sharply dropped with increasing Co^2+^ exchange (Figure 2c). A similar trend was reported by Braaten et al.,^28^ where the oxygen transport resistance of Co^2+^-doped membrane remained unchanged up to a critical Co^2+^ exchange of ∼50% and then sharply increased with increasing Co^2+^ exchange. Our results extend their findings that the cell performance (and not just the oxygen transport resistance) exhibits a similar behavior at both 100 and 50% RHs, where the performance remains relatively unaffected (up to ∼4% decrease) until the critical Co^2+^ exchange.

Mechanisms of Co^2+^-induced performance loss were assessed using a series of electrochemical analyses. The decrease in performance at Co^2+^ exchange ≥44% is accompanied by (1) an increase in sheet resistance (100% RH shown in Figure 3b) at high Co^2+^ exchange, (2) an increase in O_2_ transport loss for both RH conditions, evidenced by the larger increase in the arc at high Co^2+^ exchange in the H_2_/air EIS spectra (Figures 3c and S4) relative to a much smaller increase in the arc in the low current density EIS spectra (Figure 3d) (these results are in agreement with the trend reported by Braaten et al.^28^), and (3) an increase in kinetic resistance (Figure 3d,e). To elaborate on the reduced O_2_ transport loss, since all components were kept identical across all tests (i.e., flow channels, GDL, membrane, cathode electrode) except the Co^2+^ exchange in the ionomer, the higher O_2_ transport loss can be attributed to higher O_2_ transport resistance through the ionomer film (this topic will be further discussed in the next section). The high-frequency resistance remains relatively unaffected by the Co^2+^ (Figure 2a,b), demonstrating that the performance loss mechanisms are mainly related to mass transport (i.e., O_2_ and proton) with a much smaller contribution from kinetics only visible at the 64 and 100% Co^2+^ exchange levels.

Our experiments demonstrate that with a minimized inactive N211 membrane area, a Co^2+^ exchange of ≤44% (corresponding to 5.2 μg_Co_·cm^–2^) in the cathode can be tolerated for MEAs with a loss of only ∼4% of catalyst mass activity. O’Brien et al.^23^ reported an ∼95% Co^2+^ exchange in a Pt–Co catalyst-based electrode with a Pt loading of 0.1 mg_Pt_·cm^–2^ after 30 000 cycles of catalyst AST, well above the critical exchange limit. We also expect that the Co^2+^ effects will exacerbate with a thinner membrane (more discussion on the Cation Effects on MEAs with Increased Membrane Thickness section). Our findings demonstrate the practical relevance of Co^2+^ contamination in PEMFC electrodes and the urgent need for addressing these undesired cation contamination effects as the loss in Co will further increase during the longer operation times expected for heavy-duty applications.

### In-Plane Conductivity and Water Uptake Measurements of Cation-Doped Membranes

To further verify the cause of reduction in performance with increasing Co^2+^ doping, we characterized the in-plane proton conductivity and water uptake of membranes doped with Co^2+^. The readers are referred to the Methodology section for details of the measurement procedure.

**Figure 4:**
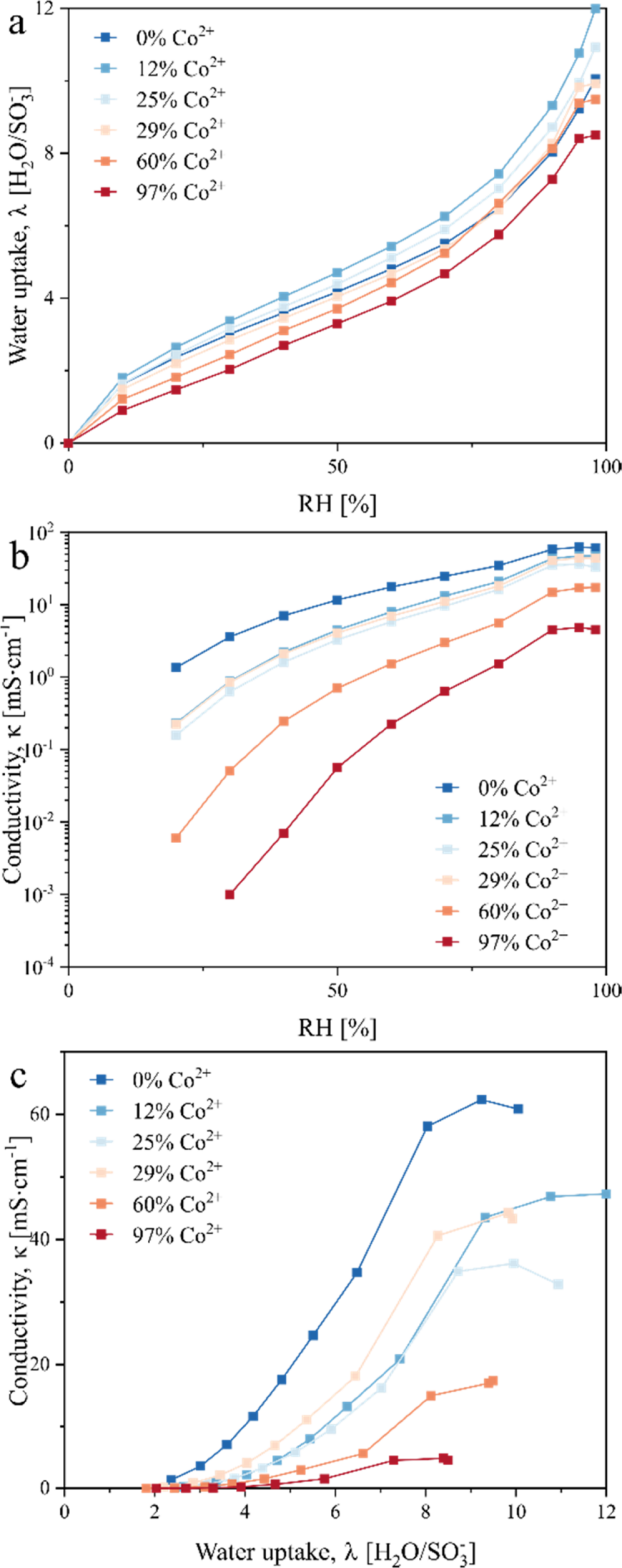
Measurement of the effect of Co^2+^ doping on PFSA properties. (a, b) The effect of Co^2+^ exchange on (a) water uptake and (b) in-plane conductivity under different RHs. (c) Conductivity plotted as a function of water uptake.

We observed strong dependence of proton conductivity and water uptake on the amount of Co^2+^ exchange (Figure 4a,b). Specifically, an increase in Co^2+^ exchange leads to lower water uptake in the electrode ionomer (except for the noncontaminated membrane, which was used as-received without any treatment), leading to more tortuous oxygen permeation pathways through the ionomer and subsequently an increase in mass transport resistance. Additionally, the proton conductivity remained similar up to ∼29% Co^2+^ exchange (noncontaminated membrane might have a higher conductivity since it was used as-received without any treatment) and then decreased with a further increase in the Co^2+^ exchange, which is in agreement with our MEA sheet resistance measurements shown in Figure 3b. As more sulfonic acid groups Co^2+^-exchange, the effective density of acid sites available for proton transport decreases, leading to an increase in sheet resistance (consistent trend across a wide range of temperatures, shown in Figure S5). Protonic conductivity decreases with an increasing Co^2+^ exchange at all water uptake levels (Figure 4c), demonstrating that the mobility of protons is hindered by the combined effects of lower water uptake and fewer sulfonic acid groups available to facilitate proton transport. Since every Co^2+^ complexing two sulfonate anions (SO_3_^–^) replaces two protons, per electroneutrality, higher cobalt fractions increase transport resistance more significantly compared to its effect on reducing the water uptake alone.

These measurements on Co^2+^-doped PFSA membranes demonstrate that Co^2+^ exchange has a significant effect on conductivity and water uptake of ionomers. However, the ionomers in the electrode exist as nanometer-scale thin films binding the catalyst sites.^33,40^ In such environments where local constraints and interactions are more dominant, ionomer films experience confinement effects that could reduce their hydration further and increase the transport resistance compared to a bulk membrane.^40−42^ Thus, the changes in ionomer properties due to Co^2+^ doping observed here for the membrane could be exacerbated in electrode ionomers, which would possibly lead to even higher transport resistances.^41,42^

### Deconvolution of Performance Loss via Impedance Modeling

**Figure 5:**
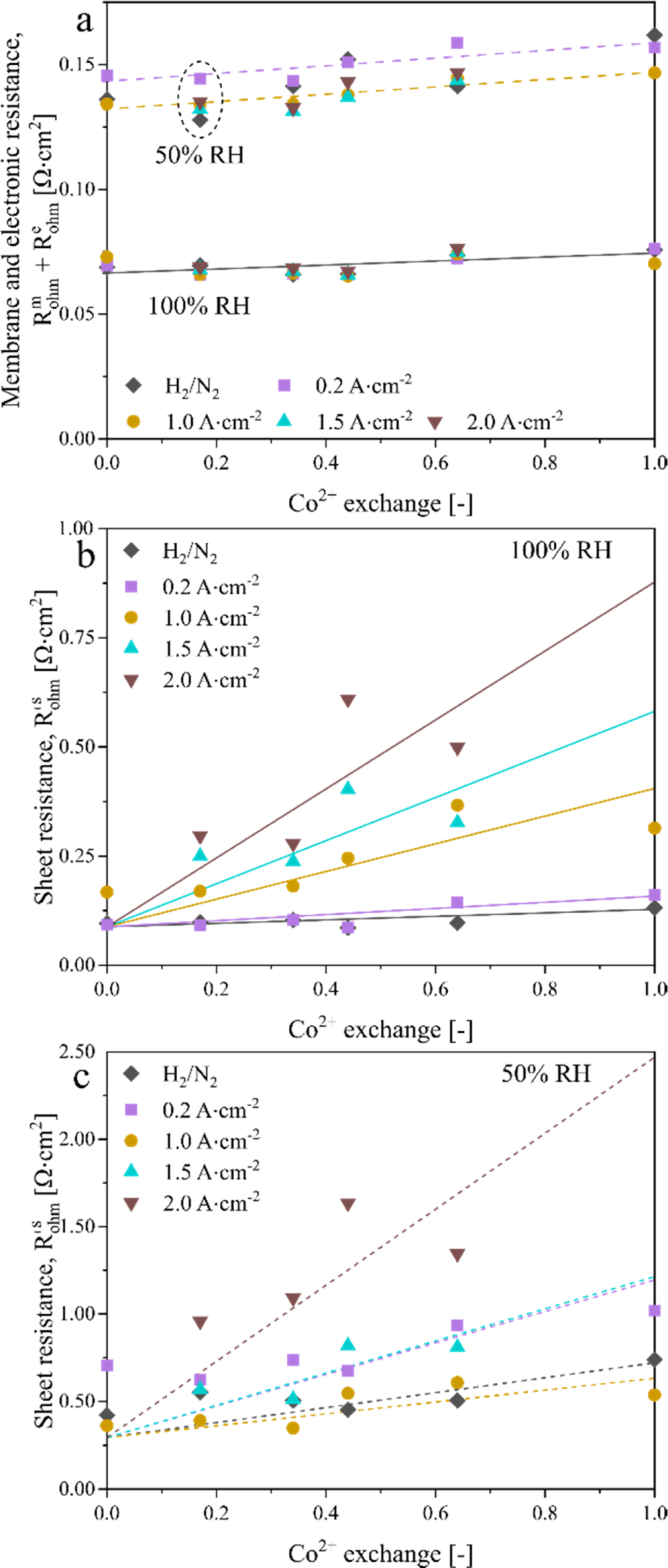
Impedance modeling to estimate the membrane and sheet resistances. (a) Membrane ohmic resistance as a function of Co^2+^ exchange at 50% RH and 100% RH. The solid lines for 100% RH and dashed lines for 50% RH have been drawn to indicate trends in symbols denoting *R*_Ω_^m^ derived from the impedance data. (b, c) Electrode sheet resistance (*R*_Ω_^s^) as a function of Co^2+^ exchange and current density at 100% RH (top) and 50% RH (bottom). The solid and dashed lines have been drawn to indicate trends in the symbol denoting *R*_Ω_^s^ derived from the impedance data.

To quantitatively compare the contribution of each loss, we break down the losses through impedance modeling. The details of the modeling procedure and the fit results are summarized in the Methodology section. Figure 5a presents the modeled high-frequency resistance (*R*_Ω_^m^*+ R*_Ω_^e^) as symbols with trend lines drawn to facilitate discussion. Here, *R*_Ω_^m^ is the proton resistance in the membrane, and *R*_Ω_^e^ is the electronic resistance. Assuming that *R*_Ω_^e^ is mainly due to the two-sided electrode/diffusion media contact resistance, taken as 36 mΩ·cm^2^, there is only a small increase in *R*_Ω_^m^ at 100% RH with Co contamination: Δ*R*_Ω_^m^ = 5.2 mΩ·cm^2^ for the highest level of Co^2+^ exchange, 100%; and Δ*R*_Ω_^m^ is almost independent of the current density. Under drier conditions such as 50% RH, *R*_Ω_^m^ generally decreases at a higher current density because of water production in the cathode and resulting in enhanced membrane water content (λ). The effect of Co contamination on *R*_Ω_^m^ is also more prominent than at 100% RH, viz., Δ*R*_Ω_^m^ = 15–18 mΩ·cm^2^ for 100% Co^2+^ exchange over 0.2–2 A·cm^–2^ current density (Figure S6 shows the change in *R*_Ω_^m^*+ R*_Ω_^e^ with current density).

We also examined the electrode sheet resistance (*R*_Ω_^s^ = NR_Ω_^si^) determined from the transmission line model (Figure 5b,c). At 100% RH, *R*_Ω_^s^ in H_2_/N_2_ (zero current density) shows a measurable increase with Co^2+^ exchange, by ∼43% for 100% Co^2+^ exchange. The increase in *R*_Ω_^s^ with Co loading is larger at a higher current density, probably because of the Co movement from the membrane to the cathode electrode. At 2 A·cm^–2^, *R*_Ω_^s^ is seen to more than quadruple with 100% Co^2+^ exchange. Figure 5c shows similar effects of Co^2+^ exchange on *R*_Ω_^s^ at 50% RH as at 100% RH. However, the dependence on current density is nonlinear because of its conflicting effects on Co^2+^ migration from the membrane to the cathode electrode and water production in the electrode. At higher current density, the electrode proton conductivity decreases because of Co^2+^ segregation in the cathode but improves because of the greater water uptake in the ionomer.

**Figure 6:**
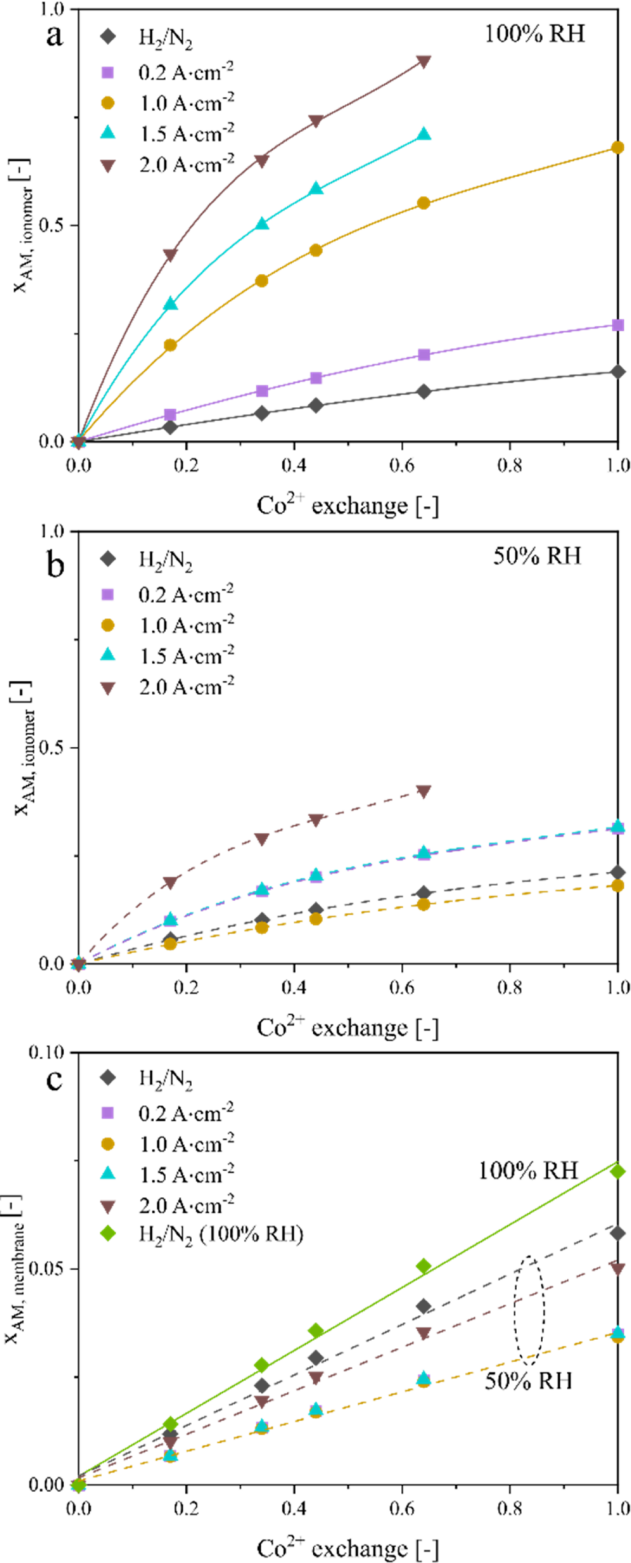
Estimation of Co^2+^ partitioning within the MEA. Co uptake in the cathode electrode at (a) 100% RH and (b) 50% RH, and (c) membrane represented by equivalent cation fraction (*x*_AM_). The trend lines are least-square fits of modeled (*x*_AM_).

Using the measured isotherms for membrane conductivity as a function of Co^2+^ exchange and water uptake (Figure 4) and assuming that these apply to ionomer as well, we have deconvoluted the data in Figure 5 to determine the Co uptake in the membrane and electrode and this is presented as equivalent cation fraction (*x*_AM_) in Figure 6. At 100% RH, Figure 6a displays a general trend of increasing *x*_AM_ in the electrode at higher current density and at higher Co^2+^ exchange. More than 50% of the sulfonic acid sites in the ionomer are occupied by Co^2+^ for the current density above 1 A·cm^–2^ and Co^2+^ exchange exceeding ∼25%. Figure 6b exhibits a different trend in *x*_AM_ in the electrode at 50% RH due to the current density affecting the ionomer water uptake (rather than the humidity of the gas stream at 100% RH), subsequently affecting Co^2+^ mobility and flux from the membrane. For the same current density and Co^2+^ exchange, *x*_AM_ is smaller at 50% RH than at 100% RH due to increased Co^2+^ migration to the electrode at a higher ionomer water uptake. Figure 6c presents complementary plots of *x*_AM_ in the membrane, as calculated from *R*_Ω_^m^. *x*_AM_ is much lower within the membrane relative to the electrode due to the larger number of sulfonic acid sites in the membrane, which also explains the negligible effect of Co^2+^ exchange on membrane resistance (Figure 5a). Overall, these results confirm the mobility of Co^2+^ and its preferential enrichment in the electrode at higher current density due to the increased potential-driven migration and higher water uptake in the ionomer.

We determined the modeled kinetic impedance (*Z*_k_) (i.e., the effective Tafel slope) and verified that *Z*_k_ does not change with Co^2+^ exchange in this study (Figure S7). While a loss in Co would result in an increased kinetic resistance in a realistic Pt–Co catalyst degradation scenario,^23^ ion exchange of Co^2+^ with the sulfonic acid groups in the ionomer had a negligible effect on the kinetic resistance. However, this contradicts the trends in the mass activity that we measured (Figure 3e), which can be attributed to lower catalyst utilization resulting from lower proton conductivity and water uptake and not the catalytic activity itself.

**Figure 7:**
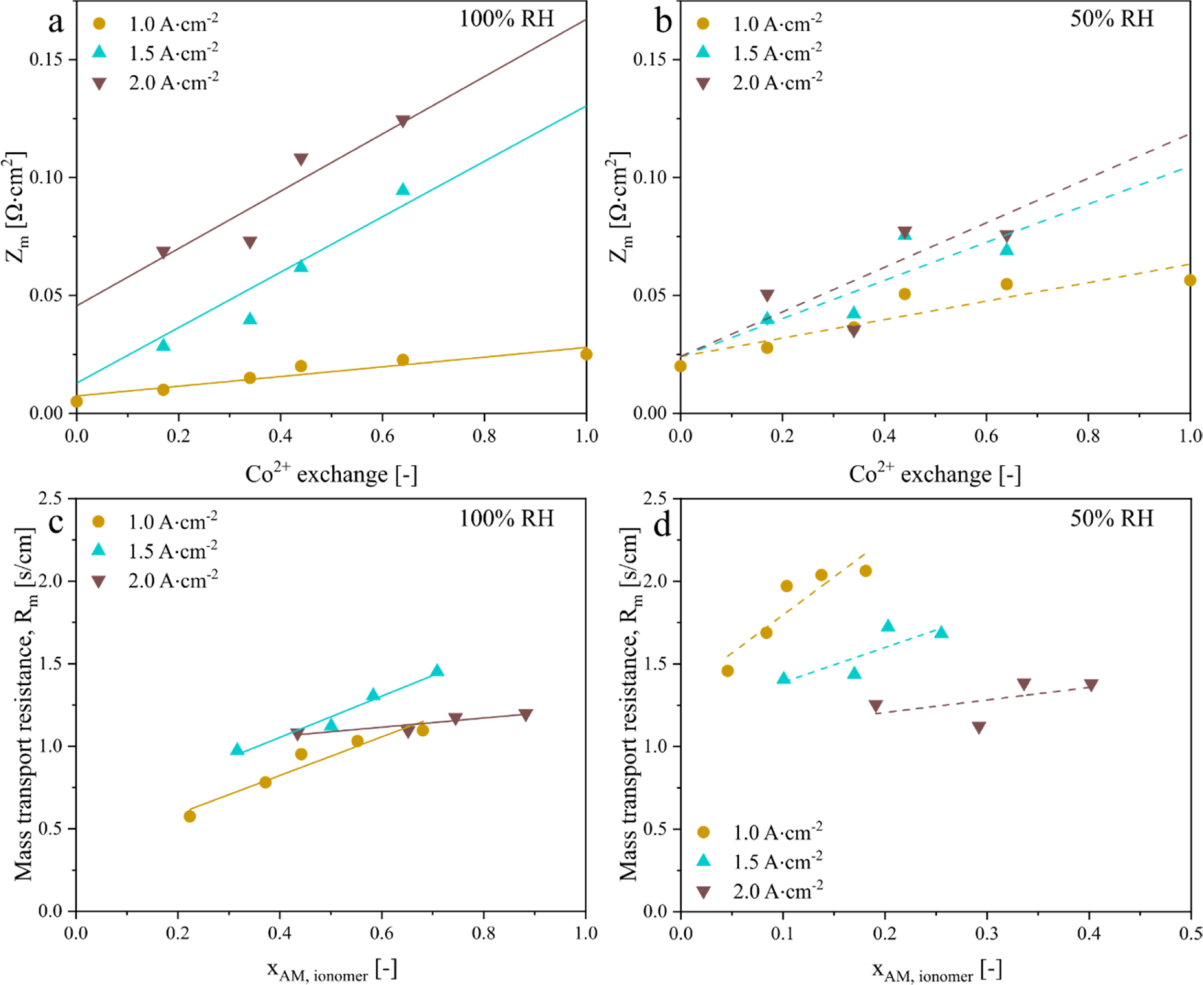
Impedance modeling to estimate the mass transport resistance. (a, b) Oxygen transport impedance (*Z*_m_) and (c, d) oxygen transport resistance (*R*_m_) derived from *Z*_m_. The solid and dashed lines have been drawn to indicate trends.

In contrast, Co^2+^ contamination strongly affects the mass transport impedance (*Z*_m_) (Figure 7a,b). At 100% RH, the modeled *Z*_m_ is also sharply higher at higher current density, which is attributed to electrode flooding. At 1 A·cm^–2^, *Z*_m_ is significantly higher at 50% RH than at 100% RH, consistent with the results from earlier studies indicating that Co^2+^ contamination of electrodes decreases O_2_ diffusivity in the ionomer, particularly under drier conditions. At 2 A·cm^–2^, however, *Z*_m_ is smaller at 50% RH than at 100% RH, likely because of reduced electrode flooding. Finally, Figure 7c,d presents the modeled mass transport resistance (*R*_m_) as a function of Co uptake and current density. At 100% RH, *R*_m_ initially increases with current density due to electrode flooding, peaks at about 1.5 A·cm^–2^, and then decreases, possibly because of the local increase in temperature. Increasing Co^2+^ exchange from 17 to 64% at 100% RH increases *R*_m_ by ∼75% at 1 A·cm^–2^ and ∼10% at 2 A·cm^–2^. At 50% RH, *R*_m_ decreases at higher current density, possibly due to the greater water production that leads to higher λ and improved O_2_ diffusivity in the ionomer. Increasing Co^2+^ exchange from 17 to 64% at 50% RH increases *R*_m_ by ∼40% at 1 A·cm^–2^ and by ∼10% at 2 A·cm^–2^.

**Figure 8:**
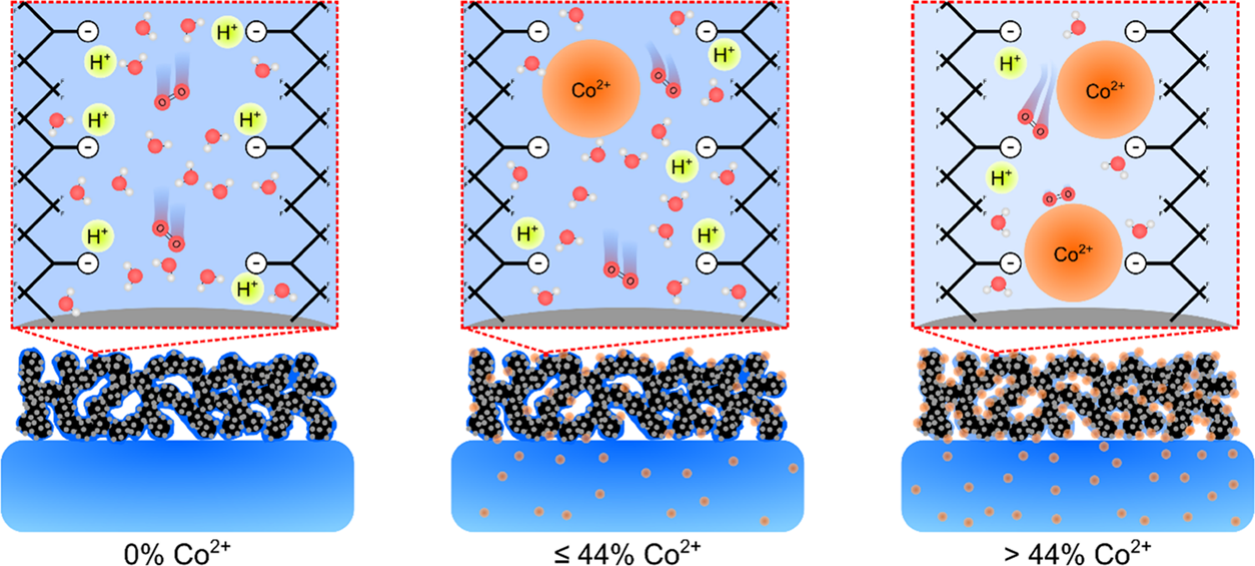
Cation contamination above critical Co^2+^ exchange influences O_2_ and H^+^ transport in the ionomer films. Sufficient water uptake and available sulfonic acid groups (indicated as “-” in the schematic) facilitate the effective transport of O_2_ and H^+^ through the ionomer films below the critical Co^2+^ exchange. However, limited water uptake and unavailable sulfonic acid groups lead to the poor transport of O_2_ and H^+^ through the ionomer films above the critical Co^2+^ exchange. The schematic is not to scale.

Overall, our modeling results demonstrate that Co^2+^ contamination has negative impacts on ionomer conductivity and oxygen transport through the ionomer (Figure 8). Impacts on the kinetics as well as membrane resistance were negligible. Specifically, although Co leaching from Pt–Co catalysts leads to reduced kinetics, the Co^2+^ contamination of the ionomer has a negligible impact on kinetics. Additionally, the amount of Co^2+^ originating from the electrode does not affect membrane resistance. We also elucidate the partitioning of Co^2+^ in between the ionomer and the membrane; at high RH, the electrode ionomer becomes heavily occupied by Co^2+^, owing to high water uptake and the subsequent increase in Co^2+^ mobility. At low RH, the Co^2+^ content in the electrode is relatively lower since the water uptake is lower and the mobility of Co^2+^ is reduced.

### Cation Effects on MEAs with Contaminated Membranes

We compared the performance between a Co^2+-^doped electrode and a Co^2+^-doped membrane to compare the results obtained from our Co^2+^ doping method with methods used in previous studies. An 8.6 cm^2^ membrane (N211) was contaminated with Co^2+^ at a loading of 9.5 μg_Co_·cm^–2^ (8%_mem_ Co^2+^), similar to the Co loading of the MEA with 100% Co^2+^-exchanged cathode (11.0 μg_Co_·cm^–2^). Although the areal Co loading was similar in the active area, the doped membrane had a higher total Co^2+^ content since the membrane area was larger than the active area (by 3.6 cm^2^).

**Figure 9:**
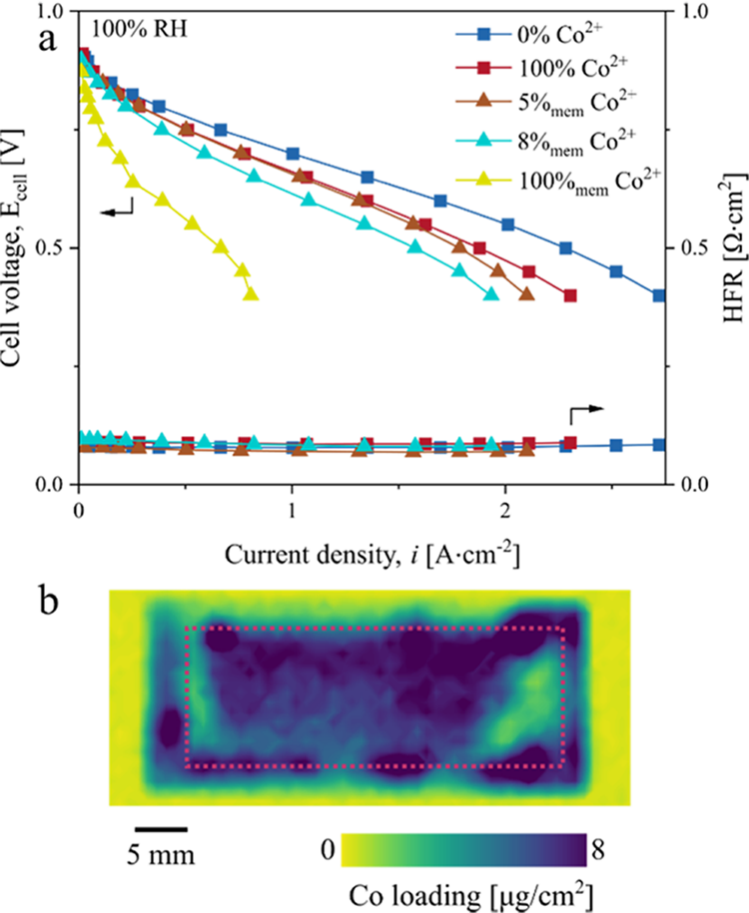
Differences in performance between the membrane and electrode doping. (a) Polarization curves of MEAs with a minimized inactive membrane area, in which either the membrane or the cathode is contaminated with Co^2+^. HFR for 100%_mem_ MEA is not indicated since the measurement was unstable (the average HFR was 0.46 Ω·cm^2^). 50% RH data are shown in Figure S8. (b) 2D XRF analysis of 8%_mem_ Co^2+^ type 2 MEA after testing. The average Co loading increased from 0 (Co^2+^ absent in the electrode initially) to 10.3 μg_Co_·cm^–2^ based on postcharacterization. The red-dashed-line rectangle indicates the active area.

Despite the slightly lower Co loading of the MEA with a doped membrane, we observed a significantly larger effect of doping on the performance; at 0.7 V and 100% RH, the current density decreased by ∼41% (Figure 9a). Since the doping of the membrane eliminates the inactive membrane area as a Co^2+^ sink, the dominant Co^2+^ migration mechanism becomes potential-driven mobility, which leads to the greater Co loading in the cathode. We verified the greater Co loading via 2D XRF measurements, which revealed a Co loading of 10.3 μg_Co_·cm^–2^ (Figure 9b), which is ∼36% higher than that of the MEA with a 100%-doped cathode electrode (i.e., 7.6 μg_Co_·cm^–2^). As an extreme case, we also examined a 100%_mem_ Co^2+^ MEA. This MEA exhibited drastically reduced performance due to the combined effects of nearly complete H^+^ displacement by Co^2+^ in both the electrode ionomer and the membrane.

We also tested an MEA in which the membrane was doped with a lower Co loading (6.2 μg_Co_·cm^–2^, 5%_mem_ Co^2+^), which was selected because it yields a Co loading in the active area similar to that of the 100% Co^2+^-exchanged electrode MEA after testing. The polarization behavior was similar to that of the 100% Co^2+^-exchanged electrode MEA (Figure 9a). We verified the Co loading after testing to be 7.5 μg_Co_·cm^–2^, which was similar to the final Co loading of the 100% Co^2+^-exchanged electrode MEA (7.6 μg_Co_·cm^–2^). Our findings demonstrate the importance of characterizing the Co loading in the active area after Co redistribution across the MEA since Co-induced performance loss is primarily driven by Co^2+^ in the active area.

### Cation Effects on MEAs with Increased Membrane Thickness

**Figure 10:**
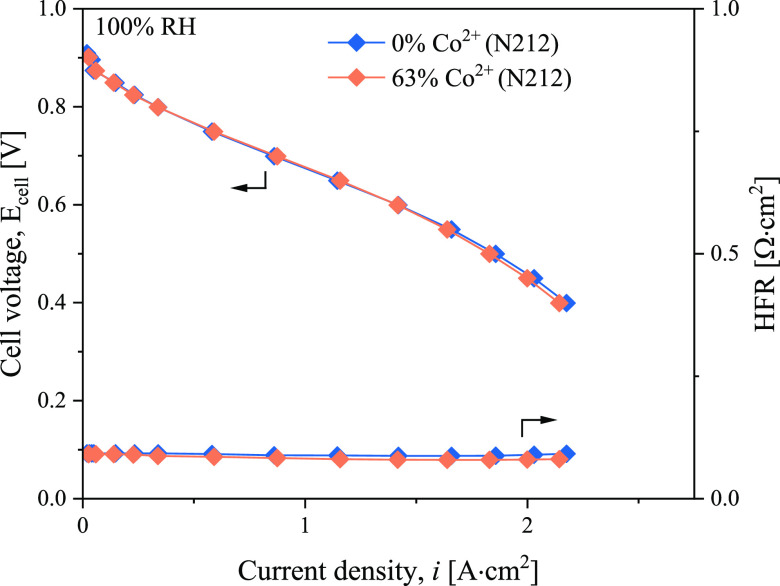
Dependence of Co^2+^ doping effect on membrane thickness. Polarization curves of MEA with an 8.6 cm^2^ membrane area, but with a thicker membrane, they reveal that, while the N211-based MEA performance is significantly hindered by Co^2+^ doping (Figure 2), the N212-based MEA performance remained relatively unchanged. This observation demonstrates that a thicker membrane provides a larger Co sink volume, allowing for higher Co^2+^ contamination levels without affecting the performance.

Our experiments with the large inactive membrane area MEA showed that the membrane acts as a Co^2+^ sink; we expect the membrane thickness to also have a strong effect on the critical Co^2+^ exchange. Indeed, we observed that the Co^2+^ doping effect became suppressed when the membrane thickness was doubled (Figure 10). While 64% of Co^2+^ exchange in the electrode for N211 led to a significant decrease in performance (Figure 2), 63% of Co^2+^ exchange in the electrode for N212 had a negligible effect. Most of the Co^2+^ was able to leave the electrode since the total Co sink volume was effectively doubled. These results have significant implications for designing next-generation MEAs; as we target thinner membranes (<10 μm) for PEMFCs with higher power density, it is important to recognize that the critical Co^2+^ exchange is expected to decrease since the Co sink volume decreases with thinner membranes. Continuing to accurately assess the effect of Co^2+^ doping will be critical to enabling a wider adoption of new materials. Unfortunately, increasing the membrane thickness (or increasing the inactive membrane area) is not a practical strategy for mitigating Co^2+^ contamination effects. A thicker membrane leads to lower power density, and a large inactive membrane is cost-ineffective. In addition, a recent membrane study^43^ showed that thickness induces slight variations in hydration, which in turn would affect the cation partitioning, necessitating additional considerations in fuel cell membrane design. Therefore, new approaches to the mitigation of Co^2+^ contamination (e.g., via new ionomers^44^) or the development of catalysts with nonleaching alloys are needed, unless the total Co that can be leached out of the catalyst is kept below the critical Co^2+^ exchange for that particular MEA design.

## Conclusions

We systematically investigated the effect of controlled cation doping of the electrode ionomer on the performance of PEMFCs. Specifically, we doped electrode decals with Co^2+^, and the electrodes were subsequently transferred onto an MEA and tested. Initially, an MEA with a large membrane area (100 cm^2^) relative to the active area (5 cm^2^) was used, and we observed Co^2+^ migrating from the electrode to the inactive membrane area. When the inactive membrane area was large relative to the active area, the inactive membrane area was observed to act as a Co^2+^ sink. Consequently, we observed a negligible effect of Co^2+^ doping on the performance when the inactive membrane area was large. We subsequently minimized the inactive area (8.6 cm^2^ membrane area) and discovered that the performance remained relatively unchanged (∼4%) up to a critical Co^2+^ exchange (∼44% in this work for a 25 μm thick membrane), with a sharp performance drop at higher exchange levels. We identified increased O_2_ and proton transport resistance as causes of the observed reduction in performance, which was further verified via ex situ measurements of Co^2+^-doped membranes. Impedance modeling showed that the proton and O_2_ transport resistances were most sensitive to Co^2+^ exchange, whereas the membrane resistance, electronic resistance, and kinetic resistance remained relatively unchanged. Additionally, we estimated the Co^2+^ partitioning between the membrane and the electrode and showed that the Co^2+^ content in the electrode generally increased with increasing current density, RH, and Co^2+^ exchange. We also observed that when the membrane was doped, the inactive membrane area no longer serves as a Co^2+^ sink, leading to higher Co^2+^ concentration in the active area. Finally, when the membrane thickness was increased, we observed that the Co^2+^ doping effects were suppressed, demonstrating that the critical exchange level is strongly dependent on the membrane thickness since the membrane acts as a cation sink. Our work demonstrates a powerful platform for accurately investigating cation contamination effects in PEMFC electrodes, which can support the design of methods to mitigate the undesired Co^2+^ contamination effects in next-generation PEMFCs, as well as other emerging electrochemical devices that may suffer from cation contamination.

## Methodology

### Cation Doping Procedure

Cathode electrodes were prepared on a decal substrate. Coated electrodes on decals were treated in CoSO_4_ solutions of different concentrations for varying times to achieve different exchange levels of cobalt ions in the decal. The electrodes were subsequently transferred onto an anode-coated membrane and conditioned under dry conditions to suppress Co^2+^ redistribution across the MEA prior to testing.

The cathode electrode decals were prepared on a polytetrafluoroethylene (PTFE) substrate. An ultrasonic spray system (ExactaCoat, Sono-Tek Corp.) was used to deposit a catalyst ink composed of ∼34 Pt wt % catalyst supported on a high-surface-area carbon (TEC10E40E, Tanaka Precious Metals), a 1000 equiv-weight (*E*_W_) ionomer dispersion (D2020, Chemours Company), and an *n*-propanol/water mixture (3:4 by volume) as a solvent. The electrode active area was 1.4 cm by 3.6 cm, the ionomer-to-carbon (I/C) ratio was 0.9, and the Pt loading was 0.25 mg_Pt_·cm^–2^, with less than 5% variation verified via spot XRF (Quant’X EDXRF, Thermo Fisher Scientific). The electrode was subsequently immersed in 20 mL of aqueous CoSO_4_ at room temperature to ion-exchange the sulfonic acid functional groups in the electrode ionomer. While previous researchers doped the membrane via a mixture of cobalt sulfate and nitric acid,^29^ we used a pure aqueous cobalt sulfate solution to eliminate the potential effects of acid treatment on the catalyst.^45^ The electrode was then immersed in deionized water for 2 h and was subsequently dried in an oven at 100 °C for 1 h. The loading of Co^2+^ in the decal was verified via spot XRF measurements. The Co^2+^ loadings in the decals were 2.5, 4.0, 5.2, 7.5, and 11.0 μg_Co_·cm^–2^, corresponding to 17, 33, 43, 63, and 100% ion exchange in the ionomer of the decal. Details on the concentration and duration used are summarized in Table 1.

**Table 1:** Concentration and Duration Used to Dope the Electrodes to Different Loadings

Co^2+^ exchange [%]	17	34	44	64	100
CoSO_4_ concentration [mM]	0.042	0.042	0.021	0.062	0.083
duration [h]	2	4	48	12	48

In addition to the controlled doping of the cathode electrode, we also doped the membrane to (1) measure the in-plane proton conductivity and water uptake at different Co^2+^ exchange levels and (2) compare the performance loss induced by doping of the membrane and electrode. We followed a previously reported method^29^ for doping the membrane (N211, Chemours Company). Specifically, the membranes were doped by immersing a 7 cm by 7 cm membrane (N211) in 150 mL of HNO_3_ and CoSO_4_ mixtures. The mixture was stirred and heated to 70 °C for 15 h. Then, the membrane was immersed in deionized water close to boiling temperature for 2 h. The membrane was finally dried on a vacuum hot plate at 100 °C. The concentration of each solution is summarized in Table 2.

**Table 2:** Concentration and Duration Used to Dope the Membranes to Different Loadings

Co^2+^ exchange [%]	5	8	25	29	60	97
CoSO_4_ concentration [M]	0.01	0.02	0.06	0.021	0.01	0.00
HNO_3_ concentration [M]	0.10	1.84	1.84	0.27	0.17	0.22

### Membrane Electrode Assembly Preparation

Two types of membrane electrode assembly (MEA) preparation methods were used, where (1) the cathode electrode was transferred by hot-pressing to a 100 cm^2^ membrane that had been coated with an anode electrode and (2) the cathode electrode decal was transferred by hot-pressing to an 8.6 cm^2^ membrane and a 6.5 cm^2^ gas diffusion electrode (GDE) (SGL 29BC, SGL carbon) as the anode. For the MEA with an 8.6 cm^2^ membrane, we placed a 100 cm^2^ and 7 μm thick Kapton sheet with the active area cut-out (1.4 cm by 3.6 cm) in between the membrane and an oversized anode GDE to ensure low gas crossover across the membrane with a minimized inactive area (3.6 cm^2^ of inactive area). The areas of the membranes with respect to the flow field are visually shown in Figure S3. For the MEA with a 100 cm^2^ membrane, we tested a 25 μm thick (N211, Chemours Company) membrane, and for the MEA with an 8.6 cm^2^ membrane, we tested both 25 and 50 μm thick (N212, Chemours Company) membranes.

The anode electrode was composed of ∼20% Pt·wt % (TEC10V20E, Tanaka Precious Metals) with a loading of 0.10 mg_Pt_·cm^–2^ and an I/C ratio of 0.5 using an identical ionomer dispersion as the cathode electrode ink. Both types of MEAs were fabricated using a cathode decal transfer by hot-pressing at ∼1900 psi, 80 °C, for 10 min. The cathode decal substrate was gently peeled off after hot-pressing to create CCMs.

### Cell Assembly and Testing Procedure

Prepared MEAs were incorporated into a single-cell PEMFC for electrochemical characterization and testing. For the details of the cell hardware used, the readers are directed to Baker et al.^46^ Polyurethane sheets were used as the gasket material, and 215 μm thick SGL 22BB (SGL Carbon) was used as the GDL (both anode and cathode GDLs for the MEA with an 8.6 cm^2^ membrane and cathode GDL for the MEA with a 100 cm^2^ membrane). For the anode GDL of the MEA built with a 100 cm^2^ membrane, a 235 μm thick GDL (SGL 29BC, SGL Carbon) was used.

The cell was tested on a commercial fuel cell test stand (850 Fuel Cell Test Station, Scribner Associates Inc.). Prior to mounting the cell, all gas lines were dried to suppress the potential Co^2+^ migration due to the presence of water. Immediately after mounting the cell, we applied 0.6 V to the cell under a dry H_2_/N_2_ anode/cathode purge while preparing for conditioning to avoid the transport of cations from the cathode electrode to the membrane.^38^ We used a modified MEA conditioning procedure that was derived from the procedure reported by General Motors.^39^ We detail the methods used to characterize the performance indicators of our fuel cells below:

(1) Polarization curves were recorded after 4 min potential holds from 0.40 to 0.95 V under 80 °C, H_2_ and air (1000 and 2000 sccm, respectively), 150 kPa, and 100 and 50% relative humidities (RHs). We also simultaneously measured the high-frequency resistance (HFR) at 5 kHz.
(2) Electrochemical impedance spectra (EIS) were collected at a 10% current perturbation from 10 kHz to 0.1 Hz under 80 °C, H_2_ and air (1000 and 2000 sccm, respectively), 150 kPa, and 100 and 50% RHs.
(3) Sheet resistances were measured at a 10 mV potential perturbation at 0.5 V from 40 kHz to 0.5 Hz under 80 °C, H_2_ and N_2_ (1000 and 2000 sccm, respectively), 150 kPa, and 100% RH.
(4) Mass activity (MA) was measured via holding the iR-corrected potential at 0.9 V under 80 °C, H_2_ and O_2_ (1000 and 2000 sccm, respectively), 150 kPa, and 100% RH for 15 min and averaging the current density over the last minute.
(5) Crossover current density was measured via constant potential hold at 0.5 V under 80 °C, H_2_ and N_2_ (1000 and 1000 sccm, respectively), 150 kPa, and 100% RH for 2 min.

We utilized a two-dimensional (2D) XRF mapping (Orbis PC Micro-XRF Analyzer, EDAX) of the MEAs to analyze the 2D Co^2+^ distribution in the MEAs or decals.

### Conductivity and Water Uptake Measurements of Co^2+^-Doped Membranes

The conductivity of cobalt-doped membranes was measured with a Scribner MTS740 with a 4-electrode in-plane conductivity probe (Scribner BT-710). Membranes were cut into strips of approximately 0.7 cm width and 2 cm length, with the width measured by a pixel count (ImageJ) and the thickness measured by a micrometer. As-doped membrane samples were loaded into the conductivity probe without further pretreatment and subjected to the following humidification profile: 2 h at 70% RH, then ramped down to 20% RH, and back up to 90% RH in 30 min increments of 10% RH. At the end of each step, a linear sweep voltammogram was collected from −0.1 to 0.1 V at 10 mV·s^–1^. Resistance was determined from the current response and used to calculate the membrane conductivity as
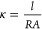
1where *l* is the interelectrode distance, *R* is the measured resistance, and *A* is the cross-sectional membrane area. The cross-sectional area at each humidity step was adjusted by the approximate volumetric swelling calculated from the water mass uptake of the membrane at the same humidity and temperature.

Mass uptake of water in membranes was measured gravimetrically during humidification using a dynamic vapor sorption instrument (DVS Advantage, Surface Measurement Systems). Membrane water uptake was measured gravimetrically as a function of relative humidity. The samples were dried in the DVS at 0% RH and 25 °C for 1 h to set a baseline mass, *M*_0_. The samples were then humidified from 0 to 90% RH with increasing RH steps of 10% and then to 95 and 98% RH at 25 °C. Samples were dehydrated back to 0% RH with the same RH values and interval but in the opposite sequence. Water (mass) uptake of the membrane, Δ*M*_W_ = *M*_W_ – *M*_0_, was continuously determined from the measured weight change. At each RH step, the sample was equilibrated until the change in its weight, Δ*M*_W_/*M*_0_, was less than 0.005%·min^–1^. The water content, λ, typically defined as the number of water molecules per sulfonate group, was calculated based on the measured water uptake Δ*M*_W_/*M*_0_^33,35^
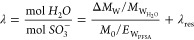
2where *M*W_H_2_O_ is the molecular weight of water (18.0 g·mol^–1^).

### Impedance Model

**Figure 11:**
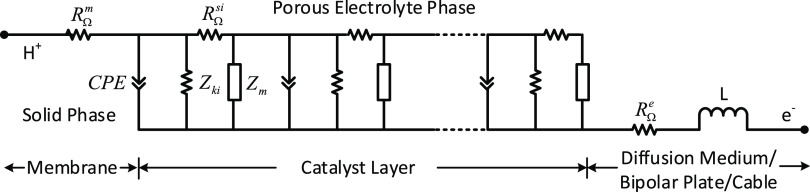
Transmission line model with the Warburg diffusion element.

**Figure 12:**
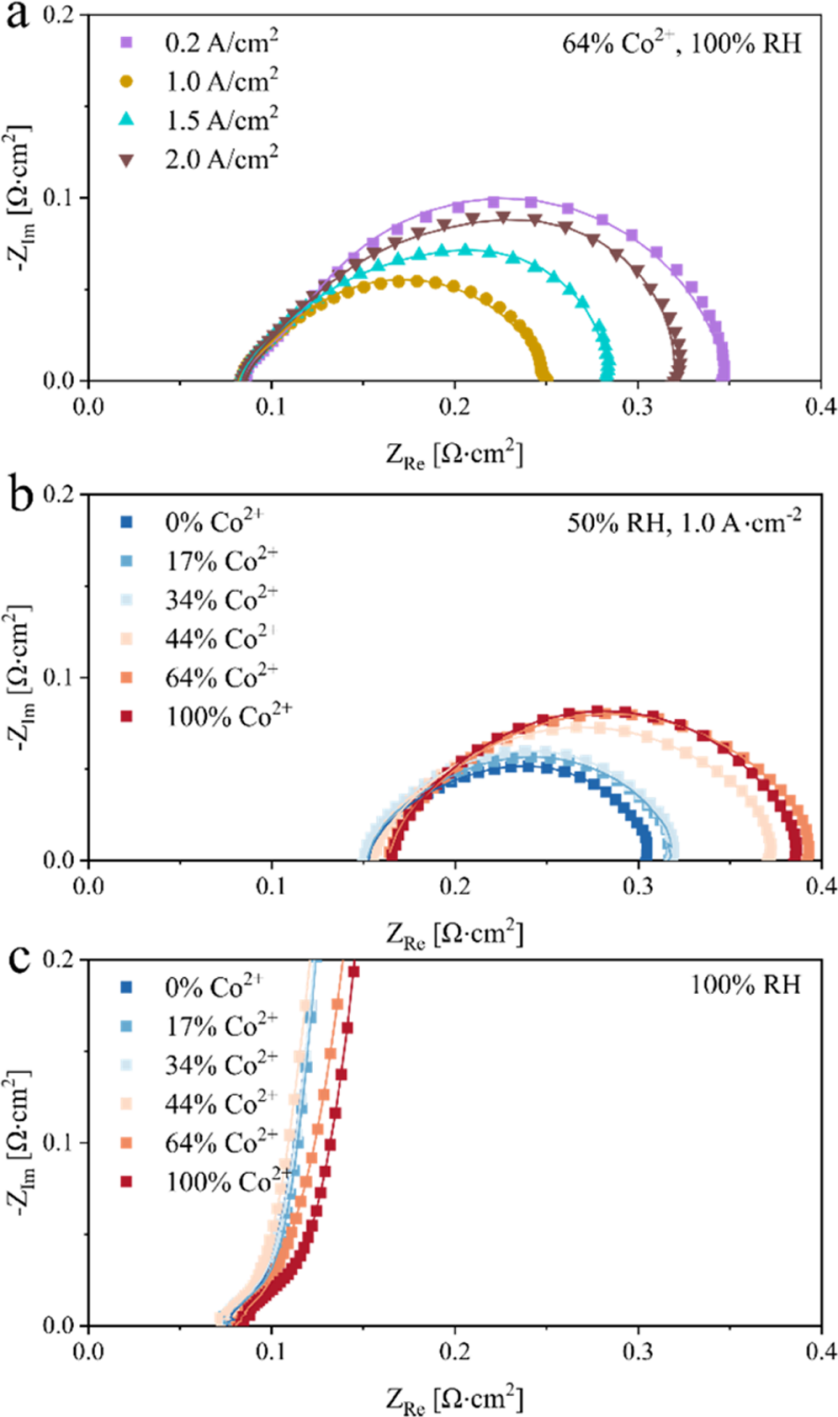
Results from the transmission line model. Model results are shown as solid lines, correlating with the measured effects of Co loading on electrode impedance in (a, b) H_2_/air and (c) H_2_/N_2_. The symbols denote the experimental data.

Following Makharia et al.,^47^ the impedance data were analyzed with the modified transmission line model in ZVIEW (see Figure 11) that includes (a) the membrane phase represented by a proton resistance element, *R*_Ω_^m^; (b) the porous electrolyte phase represented by 100 repeat units (*N*), each consisting of a double-layer capacitor element that also has a constant phase element (Warburg impedance) in parallel with a kinetic impedance element (*Z*_ki_) and both in series with an electrode resistance element (*R*_Ω_^si^); (c) a Warburg diffusion element represented by a double-layer capacitor in parallel with a Warburg resistance (*Z*_m_); and (d) the diffusion medium/bipolar/cable phase represented by the electrical resistance element (*R*_Ω_^e^) and inductance (*L*). For H_2_/N_2_ impedance with zero current density, *Z*_ki_ is set to a large number. Figure 12 compares several modeled and measured impedances in H_2_/air and H_2_/N_2_ for different current densities, relative humidities (RHs), and total Co loading. It shows that the set of values determined for the circuit elements in the transmission line model are consistent with the experimental observables in each case.

We determine the kinetic (*Z*_k_) and mass transfer (*Z*_m_) impedances from the voltage balance equation written in terms of the Nernst voltage (*E*_N_) and cathode overpotential (η_c_) at an O_2_ partial pressure at the catalyst surface () that is related to the limiting current density
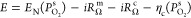
3
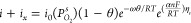
4
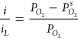
5where *i*_x_ is the crossover current density, *i*_0_ is the exchange current density, θ is the oxide coverage, ω is the activation energy, α is the symmetry factor, and *n* is the number of electrons. From the definition of impedance (*Z*_r_), it can be shown that *Z*_k_, *Z*_m_, and O_2_ transport resistances (*R*_m_) are related to η_*c*_ at an O_2_ partial pressure in the gas channel (), mass transfer overpotential (η*m*), and *i*_L_ as follows
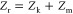
6
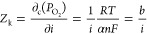
7
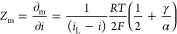
8
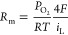
9Assuming negligible mass transport resistance at low current densities, we determined the Tafel slope, *b*, from *Z*_k_ at 0.2 A·cm^–2^ as 45 mV·dec^–1^, which is equivalent to α = 0.34 and *n* = 2. Knowing *Z*_k_, we determined *Z*_m_ at high current densities (*i* ≥ 1.0 A·cm^–2^) from the ZVIEW transmission line model with a default α of 0.25 for the ORR reaction without considering Pt oxide formation. We further determined the limiting current density using eq 8 with γ = 0.5. Finally, the mass transfer resistance *R*_m_ was determined from eq 9.

## Supporting Information Available

2D XRF maps of the MEA before electrochemical testing; polarization curves, high-frequency resistances, and impedance spectra at 50% RH; photograph of the flow field used in this study; dependence of proton conductivity on temperature; and additional impedance modeling results for membrane, electronic, and kinetic resistances (PDF)

## Abbreviations

PEMFC: proton-exchange membrane fuel cells
XRF: X-ray fluorescence
MEA: membrane electrode assembly
PFSA: perfluorosulfonic acid
