# Supplementary material for: Toward a Comprehensive Understanding of Cation Effects in Proton Exchange Membrane Fuel Cells
Source: ACS Appl Mater Interfaces. 2022 Jul 26;14(31):35555–68. doi: 10.1021/acsami.2c07085 (PMC9376928; doi:10.1021/acsami.2c07085)
Supplement: Supplementary file 1 — am2c07085_si_001.pdf [file am2c07085_si_001.pdf]

# Supporting Information

# Towards a Comprehensive Understanding of Cation Effects in Proton Exchange Membrane Fuel Cells

ChungHyuk Lee[1], Xiaohua Wang[2], Jui-Kun Peng[2], Adlai Katzenberg[3], Rajesh K. Ahluwalia[2], Ahmet Kusoglu[3], Siddharth Komini Babu[1], Jacob S. Spendelow[1], Rangachary Mukundan[1]*, Rod L. Borup[1]*

[1]Material Synthesis and Integrated Devices Group, Los Alamos National Laboratory, Los Alamos, NM, 87545, USA

[2]Energy Systems Division, Argonne National Laboratory, Argonne, IL, 60439, USA

[3]Energy Technologies Area, Lawrence Berkeley National Laboratory, Berkeley, CA, 94720, USA

*mukundan@lanl.gov; borup@lanl.gov

### *$Co^{2+}$ mobility during MEA preparation*

To ensure that the 2D XRF measurement results reported in this study are due to $Co^{2+}$ migration during testing, we performed 2D XRF measurement of a decal electrode before and after the transfer (Fig. S.1). We verified that $Co^{2+}$ does not migrate out to the inactive membrane area during the transfer, and that the Co loading was similar before and after the transfer.

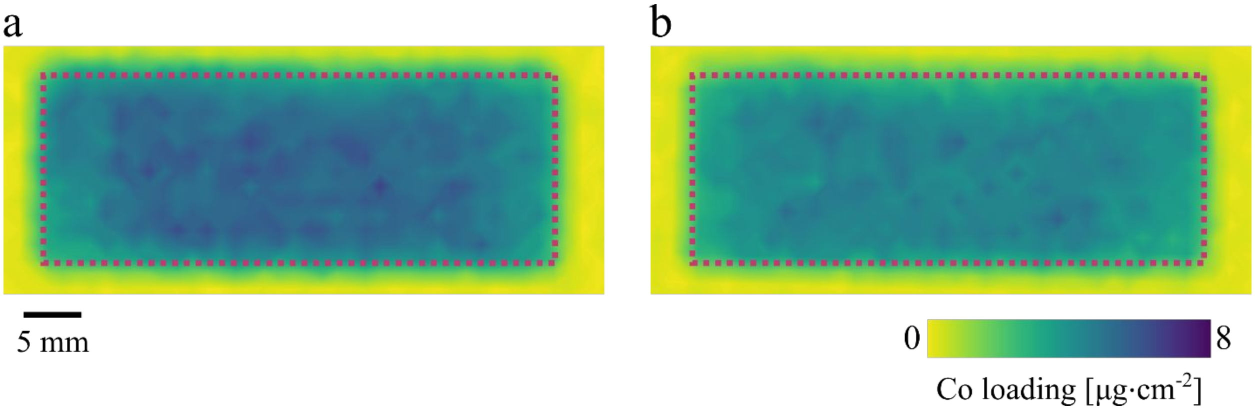


Fig. S.1: 2D XRF measurements before testing. 2D XRF measurements of the electrode (a) before and (b) after the decal transfer. The red-dashed-line rectangle indicates the active area.

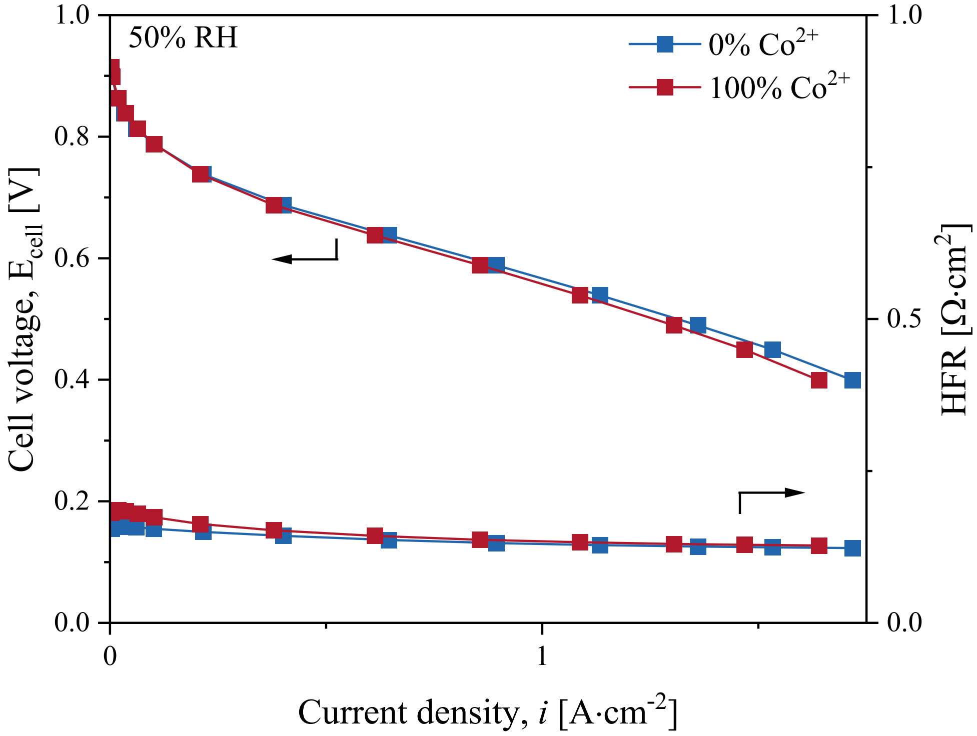


Figure S.2: $Co^{2+}$ doping effect on the performance of MEA with a large inactive membrane at 50%RH. Polarization curves of MEA with a large inactive membrane area show negligible effects of $Co^{2+}$ doping on the performance at 50% RH.

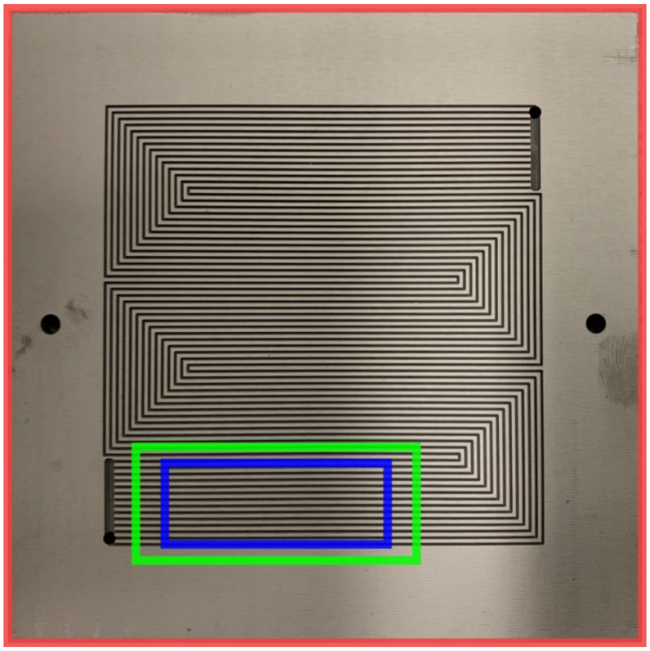

Fig. S.3: Visual aid in showing the different inactive membrane areas. Picture of the flow field; blue box indicates the active area (1.4 by 3.6 $cm^2$), red box indicates the membrane area for MEA with a large inactive membrane area (10 by 10 $cm^2$), and green box indicates the membrane area for MEA with minimized inactive membrane area (1.9 by 4.5 $cm^2$).

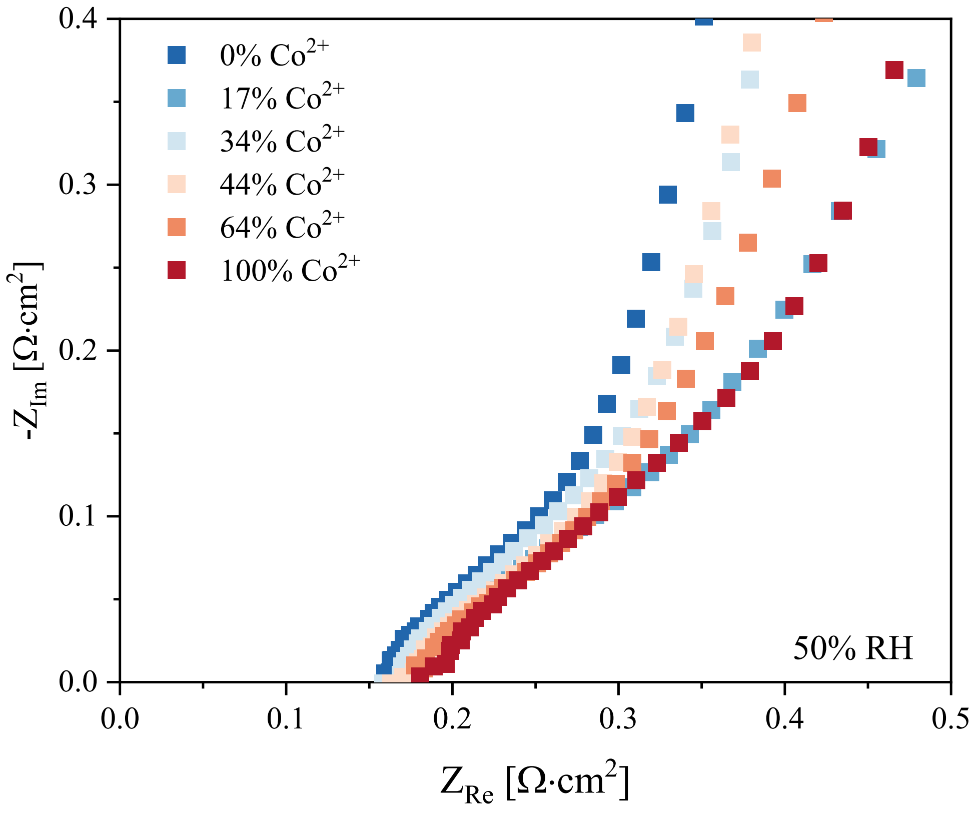


Figure S.4: $H_2/N_2$ EIS of MEA with minimized inactive membrane area at 50% RH.

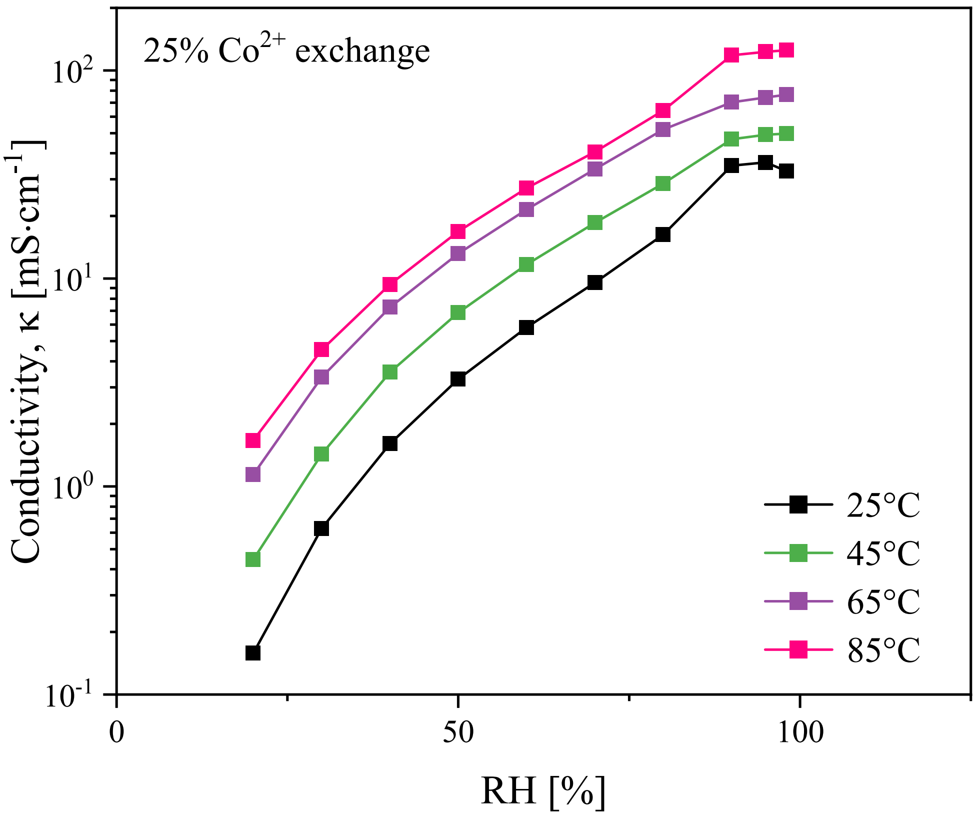


Figure S.5: The effect of temperature on the dependence of in-plane conductivity of membrane on RH, at 25% $Co^{2+}$ exchange.

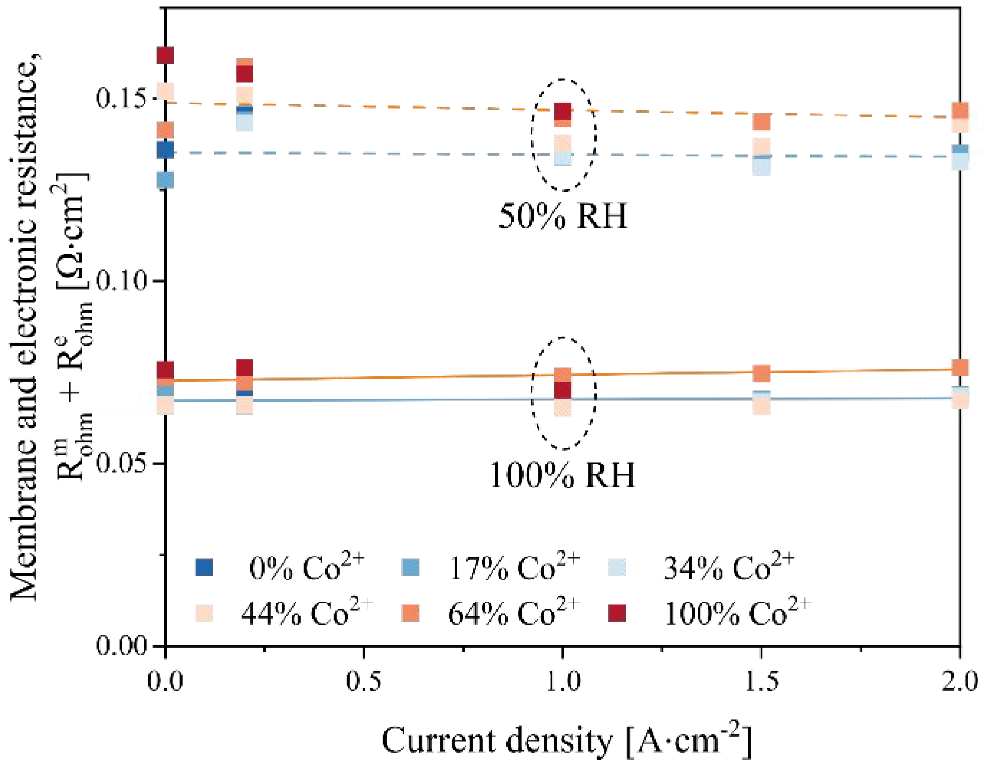


Figure S.6: Membrane Ohmic resistance as a function of current density at 50% RH and 100% RH. The solid lines for 100% RH and dashed lines for 50% RH have been drawn to indicate trends in symbols denoting $R^m_\Omega$ derived from the impedance data.

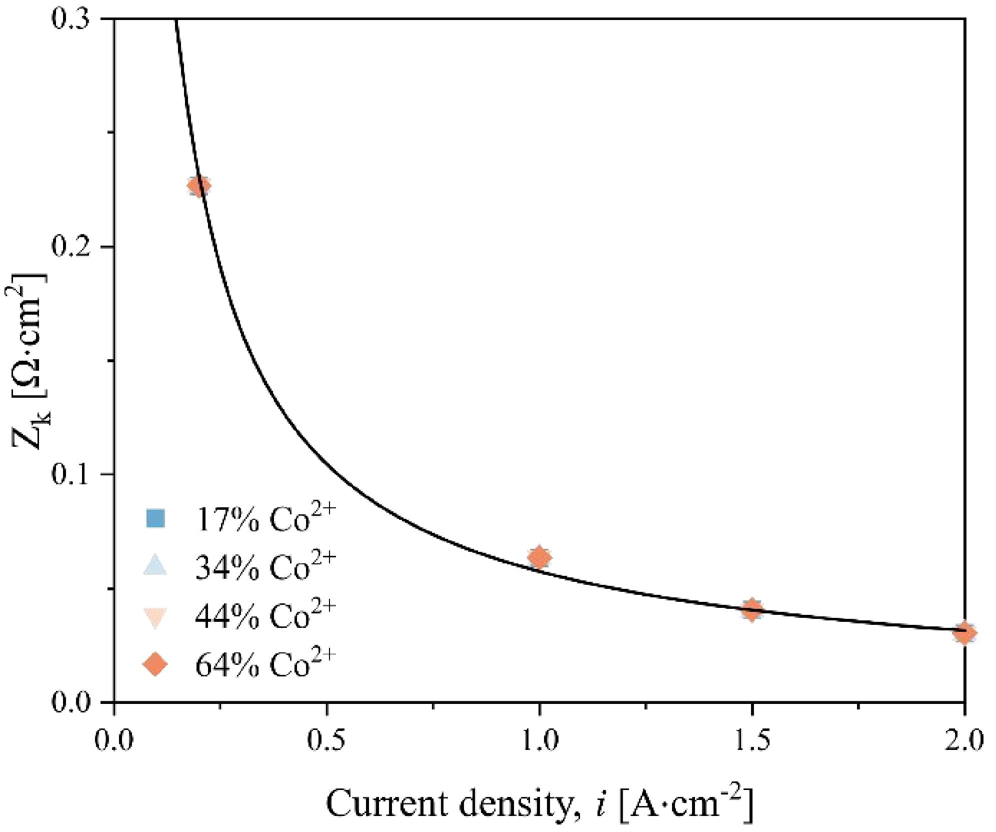


Figure S.7: Dependence of kinetic impedance ($Z_k$) on $Co^{2+}$ exchange. The kinetic resistance of the Pt/C catalyst is independent of Co doping of the ionomer.

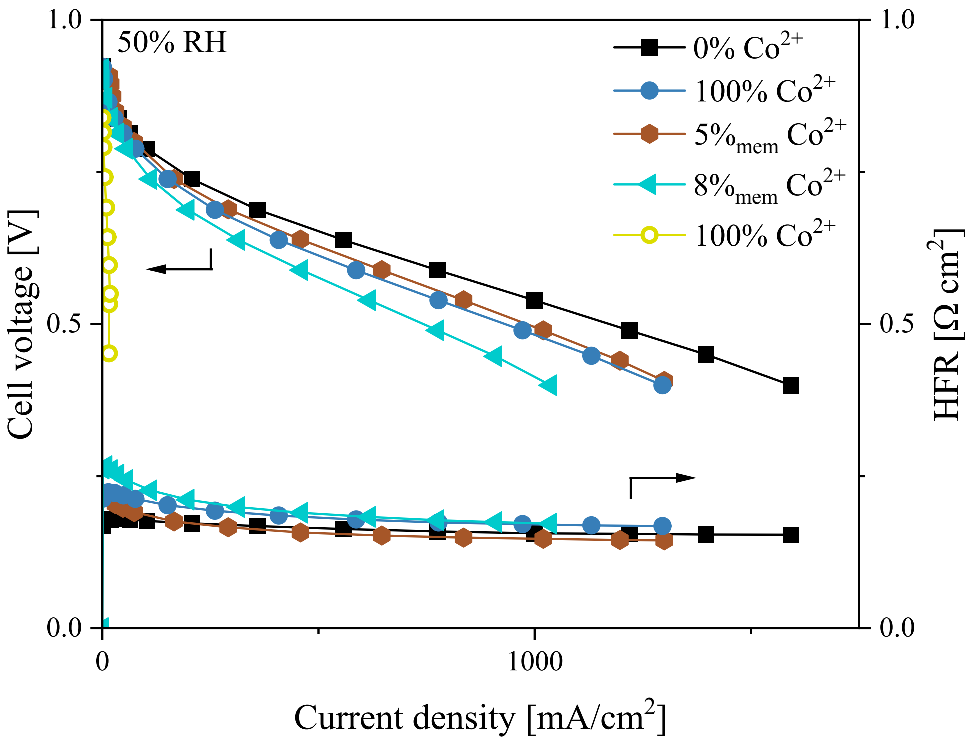


Figure S.8: Differences in performance between membrane and electrode doping at 50% RH. The doped membrane shows stronger effects of $Co^{2+}$ doping on the performance relative to the MEA with doped electrodes at 50% RH. HFR for $100\%_{membrane}$ MEA is not indicated since the measurement was unstable (the average HFR was 2.50 $\Omega \cdot cm^2$).
